# Incomplete paresis of the sciatic nerve due to massive atypical lipoma of the pelvis: a case report

**DOI:** 10.1186/1757-1626-1-296

**Published:** 2008-11-04

**Authors:** Andreas Hansch, Mieczyslaw Gajda, Joachim Boettcher, Alexander Pfeil, Werner A Kaiser

**Affiliations:** 1 Friedrich Schiller University Jena, Erlanger Allee 101, D-07747 Jena, Germany; 2 Institute of Pathology, Friedrich Schiller University Jena, Ziegelmuehlenweg 1, D-07740 Jena, Germany; 3 Institution from which the work originated: Institute of Diagnostic and Interventional Radiology, Friedrich Schiller University Jena, Germany

## Abstract

**Background:**

Liposarcomas are classified into four subtypes, with different malignancy potential and characteristic imaging appearances. Well-differentiated liposarcomas have imaging characteristics similar to those of benign lipomas, however they can be usually distinguished from lipomas because of the larger size and broader fibrous septa, with a more nodular appearance.

**Case presentation:**

This paper presents a case of atypical lipoma (well-differentiated liposarcoma) of the pelvis, leading to partial involvement of the sciatic nerve. In our case, computed tomography (CT) showed a low-density lesion. In magnetic resonance imaging (MRI), T1 and T2-weighted sequences revealed a fatty appearance with signal loss on fat saturation pulse sequences.

**Conclusion:**

The lesion was successfully resected and no other similar lesions have been found within one year of follow-up.

## Background

Liposarcoma, a malignant tumor of mesenchymal origin, is one of the most common primary neoplasms in the retroperitoneum [1]. Histologically, liposarcomas are classified in four subtypes with different potential of malignancy: well-differentiated or atypical, myxoid, pleomorphic, and round-cell subtypes. The surgical strategy and patient prognosis largely depends on these different subtypes [2].

## Case presentation

**Figure 1:**
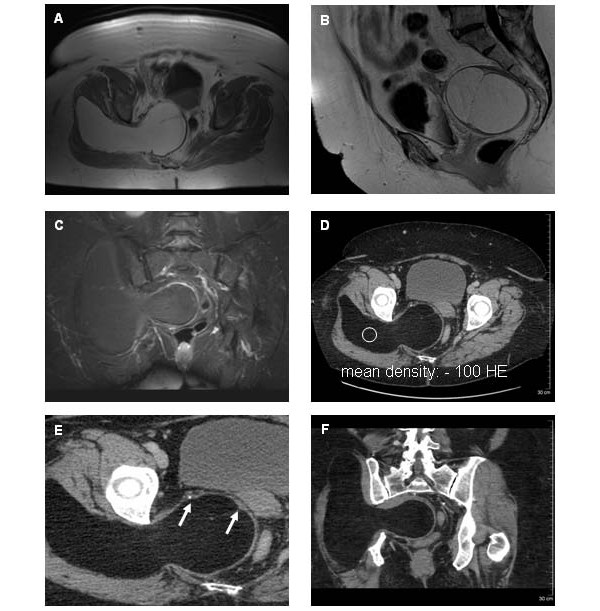
**Pelvic MR and CT imaging.** A) Massive fatty tumor with hyperintense appearance in the noncontrast T1-weighted (TR/TE, 168/5) axial image. B) The sagittal noncontrast T1-weighted (TR/TE, 600/10) image demonstrated a thin septum inside the tumor. C) The tumor showed a complete signal loss in the fat saturation sequences (TR/TE 5660/48). D) Axial noncontrast multidetector CT image demonstrated a massive soft-tissue pelvic tumor occupying the sciatic foramen. The mean density was -100HE and identified the fatty tissue characteristics. E) Magnification of the axial noncontrast multidetector CT image revealed nodular appearance of the septa (see arrows). F) Coronal reconstructed noncontrast multidetector CT image showed the expansive soft-tissue mass inside and outside the pelvic. Adjacent organs und muscles were displaced.

A 60-year old woman was admitted to the outpatient department of our hospital due to deep pain of the pelvis and distal lumbar spine. Progressive pain of the posterior right leg also developed. Neurological examination revealed a reduction of strength of the right leg and incomplete paresis of the right tibial and common peroneal nerve (both branches of the sciatic nerve). The patellar reflexes were evocable on both sides, the Achilles tendon reflex was positive on the left side but absent contralaterally. Additionally, a hypoaesthesia of the S1 dermatome was detected on the right leg. The clinical picture was therefore that of a partial impairment of the right sciatic nerve. Initially, a local irritation of the sacral spinal nerve 1 was suspected, however a magnetic resonance imaging (MRI) showed the presence of a massive tumor in the right pelvis. An extension of the MRI to the whole pelvis revealed a lipomatous appearance and the presence of septa (Figure 1A–C). The diameter of the lesion was 18 × 17 × 8 cm. The enormous tumor displaced adjacent anatomic structures, for example the right obturator and piriformis muscles were displaced to the right, the uterus and the rectum were displaced to the left. The sciatic foramen was completely occupied. The neoplasm spread out to the gluteal region, with displacement of the right gluteal muscles (minimus, medius, and maximus).

The lesion presented high signal intensities on T1-weighted sequences (Figure 1A, B) and a complete signal loss on fat-saturated T1-weighted images (Figure 1C). The fat equivalent density was verified by computed tomography (CT), with measured densities of -100 HE (Figure 1D). There was no pathological enhancement of contrast media (Figure 1E) nor any signs of bone destruction (Figure 1D–F). The CT and MRI findings, and especially the signal loss on fat-saturated T1-weighted images, indicated a lipomatous tumor, e.g. a lipoma. In addition, the huge size of the lesion and the presence of broad fibrous septa (Figure 1E) were considered indicative of a well-differentiated liposarcoma. The lesion was successfully resected.

**Figure 2:**
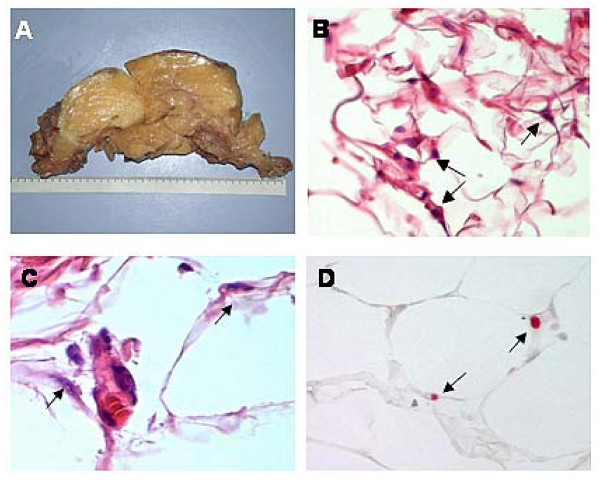
**A) Massive tumor with fatty appearance after surgery.** B) and C) Adipocytes and some atypical nuclei (see arrows, B: H&E 200× and C: H&E 400×) as a typical finding of a well-differentiated liposarcoma (atypical lipoma). D) Assessment of cell proliferation by detection of Ki67 antigen shows the low proliferation index of the atypical lipoma (see arrows, Ki67 staining 400×).

After surgical removal of the tumor, the histopathology confirmed the diagnosis of a well-differentiated liposarcoma (Figure 2). Nearly all neurological symptoms disappeared postoperatively. Only the hypoaesthesia of the S1 dermatome of the right leg remained.

## Discussion

We present a case of well-differentiated liposarcoma (atypical lipoma) in the pelvis with incomplete involvement of the sciatic nerve. About 16–18% of all malignant soft-tissue tumors in the general population are liposarcomas [3], which are of mesenchymal origin [4] and are classified into four different histological entities: well-differentiated or atypical, myxoid, pleomorphic, and round-cell subtypes [5].

Best to our knowledge, this is the first description of a case with paresis of a nerve as clinical manifestation of a lipomatous lesion. Local compression in the sciatic foramen due to the hudge size of the tumor caused impairment of the nerve.

The presented massive pelvic tumor demonstrated typical imaging findings of a lipomatous lesion. In CT imaging, the neoplasm showed a low density reaching -100 HE, except for the septa, which showed densities of soft tissue. MRI also enables a precise diagnosis of the tumor and the adjacent anatomic structures. Fat tissue demonstrates a short T1 and a relatively long T2 relaxation and therefore appears hyperintense on T1-weighted and intermediately isointense to hyperintense on T2-weighted fast-spin-echo and gradient-echo images. The presented case showed the characteristics of a benign lipoma on both CT and MR imaging [2,6]. Lipomas and well-differentiated liposarcomas consist both of lipocytes and therefore comprise a large amount of fat tissue, usually more than 75% of their volume [4]. Lipoma does not cause any symptoms. The size of the described tumor indicate an atypical lipoma. Additionally, in well-differentiated liposarcomas, however, fibrous septa are usually broader, with a more nodular appearance than seen in lipomas. If only marginally excised, atypical lipomas can recur.

Interestingly, only well-differentiated liposarcomas are predominantly fatty. The other histological subtypes usually demonstrate less than 25% or no fat tissue [2]. The most common type of liposarcoma is the myxoid one, which is considered of intermediate-grade malignancy [7]. Its signal intensities in MRI and CT imaging differ from those of lipomas and well-differentiated liposarcomas because the myxoid tumor consists of less than 10% fat tissue [7]. In CT imaging, myxoid liposarcomas often show an inhomogeneous appearance, with a density less than that of muscle tissue. In MRI, myxoid tumors demonstrate very long, homogeneous T1 and T2 relaxation times similar to those of water, low signal intensities on native T1-weighted images and high signal intensities on T2-weighted sequences [2]. However, some areas of the neoplasm consist of fat tissue with a high signal intensity on T1-weighted images and an intermediate signal intensity on T2-weighted sequences, enabling the correct diagnosis [1]. Intravenous administration of contrast media characteristically results in marked enhancement of the myxoid liposarcoma, revealing the solid nature of this tumor [4].

Pleomorphic and round cell liposarcomas are high-grade sarcomas, consisting of only small (or no) amounts of fat tissue. Both tumors show a heterogeneous appearance, also occurring in other malignant soft tissue tumors.

Preoperative diagnosis of the subtype has an impact on the surgical planning [2]. If the lesion presents a low metastatic potential (i.e., is well differentiated) a marginal excision (as performed in this case) may be possible. For high-grade tumors (round-cell or pleomorphic liposarcomas) a marginal excision is not indicated because of their high metastatic potential.

## Conclusion

In conclusion, well-differentiated liposarcomas present imaging characteristics similar to benign lipomas because both lesions consist of large amounts of fat tissue (more than 75%). However, a differentiation is usually possible because well-differentiated liposarcomas generally demonstrate a larger size and broader fibrous septa with a more nodular appearance compared to benign lipomas. Neurological symptoms as clinical manifestation of a well-differentiated liposarcoma are firstly described in this case.

## Competing interests

The authors declare that they have no competing interests.

## Authors' contributions

AH conceived the study. JB did research the literature review. AH, MG and JB prepared the manuscript. WAK edit and coordinated the manuscript.

All authors read and approved the final manuscript.

## Consent

Written informed consent was obtained from the patient for publication of this case report and accompanying images. A copy of the written consent is available for review by the Editor-in-Chief of this journal.
